# Higher education in Gerodontology in European Universities

**DOI:** 10.1186/s12903-017-0362-9

**Published:** 2017-03-28

**Authors:** Anastassia Kossioni, Gerry McKenna, Frauke Müller, Martin Schimmel, Jacques Vanobbergen

**Affiliations:** 1 Division of Gerodontology, Department of Prosthodontics, School of Dentistry, National and Kapodistrian University of Athens, Thivon 2 Goudi, Athens, 11527 Greece; 2 Centre for Public Health, Royal Victoria Hospital, Queens University Belfast, Belfast, Northern Ireland; 3 Department of Gerodontology & Removable Prosthodontics, University of Geneva, Geneva, Switzerland; 4 Division of Gerodontology, School of Dental Medicine, University of Bern, Bern, Switzerland; 5 Department of Community Dentistry, Ghent University, Ghent, Belgium

**Keywords:** Geriatric dentistry (gerodontology), Dental education, European dental schools

## Abstract

**Background:**

The rapid aging of the European population and the subsequent increase in the oral care needs in older adults necessitates adequate training of dental professionals in Gerodontology (Geriatric Dentistry). This study was designed to investigate the current status of Gerodontology teaching amongst European dental schools at the undergraduate, postgraduate and continuing education levels.

**Methods:**

An electronic questionnaire was developed by a panel of experts and emailed to the Deans or other contact persons of 216 dental schools across 39 European countries. The questionnaire recorded activity levels, contents and methodology of Gerodontology teaching as part of dental education programs. Repeated e-mail reminders and telephone calls were used to encourage non-responders to complete the questionnaire.

**Results:**

A total of 123 responses from 29 countries were received (response rate: 56.9%). Gerodontology was taught in 86.2% of schools at the undergraduate level, in 30.9% at the postgraduate level and in 30.1% at the continuing education level. A total of 43.9% of the responding schools had a dedicated Gerodontology program director. Gerodontology was taught as an independent subject in 37.4% of the respondent schools. Medical problems in old age, salivary impairment and prosthodontic management were the most commonly covered topics in Gerodontology teaching. Clinical teaching took place in 64.2% of the respondent schools, with 26.8% offering clinical training in outreach facilities.

**Conclusions:**

The vast majority of European dental schools currently teach Gerodontology at the undergraduate level. More training opportunities in oral care of frail elders should be offered, and more emphasis should be placed on interdisciplinary and interprofessional training, educational collaborations, and the use of modern technologies. Dedicated postgraduate Gerodontology courses need to be developed to create a significant number of specialized dentists and trained academics.

**Electronic supplementary material:**

The online version of this article (doi:10.1186/s12903-017-0362-9) contains supplementary material, which is available to authorized users.

## Background

Europe is the continent with the highest proportion of older population in the world. In 2015, 17.6% of the Europeans were over 65 years of age compared to 14.7% in 2000 [1]. This proportion will further increase to 28.4% by 2060 [1]. In 2016, in the EU-28 countries, almost 18.9% of the total population was over 65 years and 5.3% over 80 years of age [2]. An increasing proportion of the older population in Europe is retaining a number of natural teeth, often heavily restored and periodontally compromized, which inevitably leads to an increase in the prevalence of chronic oral diseases and oral care needs [3–7]. Compromized general health, poor physical and cognitive function, ineffective and irregular oral hygiene habits, unhealthy dietary habits, are all contributing factors to poor oral health in this population. Additional public-health related modifiers of oral health in older adults are economic pressures on existing health care systems, as well as inadequate access to preventative dental care [3, 8–11].

In 2009, the European College of Gerodontology (ECG) published the “undergraduate curriculum guidelines in Gerodontology” [12], in order to guide curriculum development in dental schools throughout Europe, as a result of significant demographic transition. The ECG guidelines have been included in the document on the “Profile and competences for the graduating European dentist-update 2009” published by the Association for Dental Education in Europe (ADEE), aiming to guide dental education in Europe [13]. Moreover, in 2016, the Council of the European Dentists (CED) recommended the inclusion of Gerodontology to the European Commission, as part of the revised compulsory study program for dental practitioners in Europe.

However, as dental curricula in Europe are mainly directed by national bodies or at university level, Gerodontology programs may vary widely amongst European dental schools. Following the publication of the ECG undergraduate curriculum guidelines and the educational recommendations by the ADEE, this study was designed to investigate the current status of Gerodontology teaching amongst European dental schools at the undergraduate, postgraduate and continuing education levels.

## Methods

An electronic questionnaire in English, including closed and open-ended questions, was developed by a panel of experts from the ECG. The pdf format of the electronic questionnaire is presented in Additional file 1. The questions were based on previously applied questionnaires to facilitate comparisons [14–17] and consisted of 52 questions. The items were grouped into five categories: a) basic demographic information on the dental school, b) the prevalence of any dentists specializing in Gerodontology in the country, c) undergraduate education in Gerodontology, d) postgraduate education in Gerodontology, e) continuing education/continuing professional development (CE/CPD) in Gerodontology, and f) developing of educational material. All closed-ended questions offered the option to add more details where appropriate. Basic and more detailed information was requested on the undergraduate Gerodontology curriculum for dental studies, including current activity levels; program structure; assignment of a specific program director; qualifications of teaching staff; subject content; educational methodology; and educational material used. The subsequent section of the questionnaire was dedicated to postgraduate education where information on the content and structure of programs in Gerodontology was requested. A further section was dedicated to any continuing education courses (CE/CPD) organized by the dental schools/universities. Finally, the participants were asked to detail if any educational material in the domain of Gerodontology was published in their country. The questionnaire was pilot-tested in a focus group of five Gerodontology educators from different countries and was refined and finalized by the expert team.

In January 2016, an introductory email, including a hyperlink to the questionnaire, was sent to the Deans and other contact persons of 216 dental schools in 39 European countries. The Deans were asked to either answer the survey themselves or forward the link to the appropriate faculty member with knowledge on Gerodontology teaching at their school. The mailing list included all European dental schools. Transcontinental countries (Russia, Turkey, Azerbaijan, and Kazakhstan) were excluded, and two EU member countries (Cyprus and Luxemburg) were also excluded as they did not have a dental school at the time of the investigation. The list of dental schools was obtained from a web-search; consultation with the published lists of the ADEE and other European scientific associations; communication with ECG members and dental academics known to the researchers; and consultation with local learned societies.

A multi-mode approach to increase response rates has been applied. Repeated e-mail reminders or telephone calls were used for non-respondents and personal networks were exploited to identify potential contact persons. The survey was also advertized in the ECG meeting in Paris (June 2016), where representatives of the non-responding schools or countries were approached by the researchers.

### Statistical analysis

The data collected was summarized and analyzed using descriptive statistics. Percentages were calculated based on the number of responding schools.

## Results

A total of 123 responses from 29 countries were received by September 2016, equivalent to a response rate of 56.9% for all schools, and 58.8% for the schools in the EU-28 countries.

The response rate was 100% for 14 countries: Croatia, Denmark, Estonia, Finland, former Yugoslav Republic of Macedonia (FYROM), Greece, Iceland, Ireland, Lithuania, Malta, Norway, Moldavia, Switzerland and the Netherlands (Table 1). None of the dental schools in Albania, Andorra, Armenia, Belarus, Bosnia and Herzegovina, Bulgaria, Czech Republic, Latvia, Montenegro and Slovenia replied. Low response rates (<50%) were recorded for Ukraine (12.5%), Italy (31.4%), Belgium (40%), Romania (40%), Poland (40%), and Portugal (42.9%).

**Table 1:** The list of schools participating in the survey

	Country	Number of schools contacted	Number of schools responding (%)	Population over 65 years in 2015 (%) [1]
1	Albania	1	0 (0.0)	12.4
2	Andorra	1	0 (0.0)	Not reported
3	Armenia	1	0 (0.0)	10.8
4	Austria	4	3 (75.0)	18.8
5	Belarus	2	0 (0.0)	14.0
6	Belgium	5	2 (40.0)	18.2
7	Bosnia and Herzegovina	1	0 (0.0)	15.4
8	Bulgaria	3	0 (0.0)	20.0
9	Croatia	3	3 (100.0)	18.9
10	Czech Republic	4	0 (0.0)	18.1
11	Denmark	2	2 (100.0)	19.0
12	Estonia	1	1 (100.0)	18.8
13	Finland	4	4 (100.0)	20.5
14	France	16	12 (75.0)	19.1
15	FYROM	1	1 (100.0)	12.3
16	Germany	29	25 (86.2)	21.2
17	Greece	2	2 (100.0)	21.4
18	Hungary	4	2 (50.0)	17.8
19	Iceland	1	1 (100.0)	13.7
20	Ireland	2	2 (100.0)	13.1
21	Italy	35	11 (31.4)	22.4
22	Latvia	1	0 (0.0)	19.4
23	Lithuania	2	2 (100.0)	18.8
24	Malta	1	1 (100.0)	19.2
25	Montenegro	1	0 (0.0)	13.6
26	Norway	3	3 (100.0)	16.3
27	Poland	10	4 (40.0)	15.5
28	Portugal	7	3 (42.9)	20.8
29	Moldavia	1	1 (100.0)	10.0
30	Romania	10	4 (40.0)	17.3
31	Serbia	4	2 (50.0)	17.1
32	Slovakia	2	1 (50.0)	13.8
33	Slovenia	1	0 (0.0)	18.0
34	Spain	15	10 (66.7)	18.8
35	Sweden	4	3 (75.0)	19.9
36	Switzerland	4	4 (100.0)	18.0
37	The Netherlands	3	3 (100.0)	18.2
38	United Kingdom	17	10 (58.8)	17.8
39	Ukraine	8	1 (12.5)	15.3
	Total	216	123 (56.9)	

### Schools’ demographics

The majority of dental schools who completed the questionnaire were publically funded (113, 91.9% of the respondents). Seventy-four schools (60.1%) reported a five-year undergraduate curriculum whilst a smaller number ran a six-year undergraduate program (40 schools, 32.5%). It should be noted that most European undergraduate dental curricula consist of 10 semesters, while smaller numbers of schools offer 11 or 12 semesters study programs. A total of 25 different official teaching languages/dialects were reported. Some schools, mainly in Eastern Europe, offered programs in two or even three different languages for international students.

### Dentists specializing in gerodontology

Forty-one (33.3%) of the respondents reported that there were dentists specializing in Gerodontology in their country, whilst national Gerodontology Scientific Associations existed in 13 countries.

### Undergraduate gerodontology education

Gerodontology was taught as a subject in 106 out of the 123 schools (86.2% of the respondents). In eleven countries with a 100% response rate, Croatia, Denmark, Estonia, Finland, FYROM, Greece, Ireland, Malta, Norway, Switzerland, and the Netherlands, all dental schools teach Gerodontology. Other countries, with lower response rates, where all respondents teach gerodontology include Belgium, Hungary, Poland, Serbia, Slovakia, Sweden, UK and Ukraine.

**Fig. 1:**
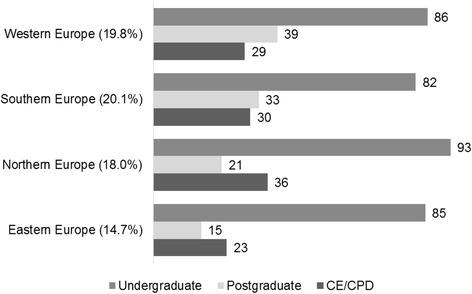
Prevalence of undergraduate, postgraduate and continuing dental education/continuing professional development (CE/CPD) in Gerodontology by geographical area (%). Percentages are calculated based on the number of responding schools per geographical area. The numbers in parentheses refer to the percentage of the total population over 65 years of age in each of the geographical areas [1]

Figure 1 illustrates the prevalence of undergraduate teaching by geographical area. Eastern Europe includes Armenia, Belarus, Bulgaria, Czech Republic, Hungary, Poland, Moldavia, Romania, Slovakia, and Ukraine [1]. Northern Europe includes Denmark, Estonia, Finland, Iceland, Ireland, Latvia, Lithuania, Norway, Sweden and the UK [1]. Southern Europe includes Albania, Andorra, Bosnia and Herzegovina, Croatia, Greece, Italy, Malta, Montenegro, Portugal, Serbia, Slovenia, Spain and former Yugoslav Republic of Macedonia (FYROM) [1]. Western Europe includes Austria, Belgium, France, Germany, Luxembourg, Netherlands and Switzerland [1]. The highest prevalence was reported in Northern European countries (26 out of 28 schools, 92.9%) and the lowest in Southern Europe (27 out of 33 schools, 81.8%).

Of those schools who did not teach Gerodontology at the time of the survey (*n* = 17), 8 (47.1%) reported that they were considering incorporating a Gerodontology course in the near future.

Gerodontology was taught as an independent subject in 46 schools (37.4% of the respondents) and was a compulsory element in 64 schools (52%) and partially in a further 19 schools (15.5%). When embedded in other disciplines, these were mainly Prosthodontics (38 schools, 30.9%). Other disciplines that included Gerodontology teaching were Preventive and Community Dentistry (22 schools, 17.9%), Special Care Dentistry (20 schools, 16.2%), Operative Dentistry (20 schools, 16.2%), Periodontology (17 schools, 13.8%), and to a lesser extent Oral and Maxillofacial Surgery (8 schools, 6.5%) and Endodontics (8 schools, 6.5%).

The majority of schools (70, 56.9%) offered Gerodontology within higher semesters (7^th^-12^th^ depending on the study program). Five schools (4.1%) offered Gerodontology only in the first semesters (1^st^-5^th^). Twenty-two (17.9%) schools offered Gerodontology throughout the different years of the curriculum (usually five or six), starting from the 1^st^ semester.

Fifty-four schools (43.9% of the respondents) had a dedicated program director, and 21 schools (17.1% of the respondents) had a specific Gerodontology department. Twenty-five out of the 54 (46.3%) program directors had themselves undertaken specific training in Gerodontology or Special Care Dentistry.

The majority of staff teaching Gerodontology were prosthodontists (78 schools, 63.4%) whilst others came from other oral disciplines. Thirty-three schools (26.8%) reported that they offer interdisciplinary courses including dentists, physicians (25 schools, 30.2%), psychologists (12 schools, 9.8%), nurses (6 schools, 4.9%), dental hygienists (2 schools, 1.6%), social workers (1 school, 0.8%) and sociologists (1 school, 0.8%).

The main theoretical topics in undergraduate Gerodontology teaching are presented in Table 2. There was a significant agreement (>60%) in many topics amongst the European dental schools including medical problems in old age; salivary impairment; denture related conditions and prosthodontic management in old age; demographics; caries risk assessment; age-changes of the orofacial system; and epidemiology of oral health in the elderly population.

**Table 2:** Teaching topics in undergraduate Gerodontology curricula in selected countries with high response rates (>70%) and in all respondent schools

Theoretical topics in Gerodontology teaching	Austria n (%)^a^	Croatia n (%)^a^	Denmark n (%)^a^	Finland n (%)^a^	France n (%)^a^	Germany n (%)^a^	Greece n (%)^a^	Ireland n (%)^a^	Lithuania n (%)^a^	Norway n (%)^a^	Sweden n (%)^a^	Switzerland n (%)^a^	Netherlands n (%)^a^	Total number of schools n (%)^b^
Medical problems in old age	1 (33.3)	2 (66.6)	2 (100)	4 (100)	6 (50)	12 (48)	2 (100)	2 (100)	1 (50)	3 (100)	2 (66.6)	4 (100)	3 (100)	87 (70.7)
Salivary impairment/xerostomia	1 (33.3)	3 (100)	2 (100)	4 (100)	6 (50)	10 (40)	2 (100)	2 (100)	1 (50)	3 (100)	2 (66.6)	4 (100)	3 (100)	79 (64.2)
Denture related conditions and prosthodontic management	2 (66.6)	3 (100)	0 (0)	4 (100)	5 (41.7)	14 (56)	2 (100)	2 (100)	1 (50)	3 (100)	2 (66.6)	4 (100)	3 (100)	79 (64.2)
Demographics	1 (33.3)	2 (66.6)	2 (100)	4 (100)	6 (50)	15 (60)	2 (100)	2 (100)	1 (50)	3 (100)	2 (66.6)	4 (100)	3 (100)	76 (61.8)
Caries risk assessment	1 (33.3)	3 (100)	1 (50)	4 (100)	6 (50)	11 (44)	2 (100)	2 (100)	1 (50)	3 (100)	2 (66.6)	4 (100)	3 (100)	76 (61.8)
Age-changes of the orofacial system	1 (33.3)	3 (100)	1 (50)	4 (100)	6 (50)	12 (48)	2 (100)	2 (100)	1 (50)	3 (100)	2 (66.6)	4 (100)	2 (66.6)	75 (61.0)
Epidemiology of oral health	1 (33.3)	3 (100)	2 (100)	4 (100)	6 (50)	11 (44)	2 (100)	2 (100)	1 (50)	3 (100)	2 (66.6)	4 (100)	2 (66.6)	74 (60.2)
Biology physiology psychology of ageing	1 (33.3)	2 (66.6)	2 (100)	3 (75)	5 (41.7)	12 (48)	2 (100)	1 (50)	1 (50)	3 (100)	2 (66.6)	4 (100)	2 (66.6)	71 (57.7)
Nutritional/chewing problems	1 (33.3)	2 (66.6)	1 (50)	4 (100)	6 (50)	11 (44)	1 (50)	2 (100)	0 (0)	3 (100)	1 (33.3)	4 (100)	3 (100)	69 (56.1)
Periodontal disease	1 (33.3)	3 (100)	1 (50)	4 (100)	7 (58.3)	8 (32)	1 (50)	1 (50)	1 (50)	2 (66.6)	2 (66.6)	1 (25)	2 (66.6)	69 (56.1)
Oral mucosal diseases	1 (33.3)	3 (100)	1 (50)	3 (75)	6 (50)	8 (32)	1 (50)	2 (100)	1 (50)	3 (100)	2 (66.6)	2 (50)	3 (100)	69 (56.1)
Pharmacology and polypharmacy	1 (33.3)	2 (66.6)	2 (100)	4 (100)	6 (50)	10 (40)	2 (100)	1 (50)	1 (50)	3 (100)	1 (33.3)	4 (100)	3 (100)	68 (55.3)
Association between general and oral health	1 (33.3)	2 (66.6)	2 (100)	4 (100)	5 (41.7)	7 (28)	2 (100)	1 (50)	1 (50)	3 (100)	2 (66.6)	4 (100)	2 (66.6)	68 (55.3)
Appropriate management of oral and dental conditions for each patient according to the individual needs and demands	1 (33.3)	1 (33.3)	1 (50)	4 (100)	5 (41.7)	10 (40)	2 (100)	1 (50)	0 (0)	3 (100)	2 (66.6)	4 (100)	2 (66.6)	61 (49.6)
Management of people with compromised health and various levels of dependency	1 (33.3)	2 (66.6)	1 (50)	4 (100)	4 (33.3)	8 (32)	2 (100)	1 (50)	0 (0)	3 (100)	2 (66.6)	4 (100)	3 (100)	59 (48)
Risk assessment	1 (33.3)	2 (66.6)	1 (50)	4 (100)	6 (50)	5 (20)	2 (100)	1 (50)	1 (50)	3 (100)	2 (66.6)	4 (100)	3 (100)	58 (47.2)
Barriers to oral care	1 (33.3)	1 (33.3)	1 (50)	4 (100)	3 (25)	7 (28)	1 (50)	2 (100)	0 (0)	3 (100)	2 (66.6)	4 (100)	3 (100)	56 (45.5)
Tooth wear assessment	1 (33.3)	3 (100)	0 (0)	2 (50)	4 (33.3)	7 (28)	1 (50)	0 (0)	0 (0)	3 (100)	0 (0)	1 (25)	3 (100)	55 (44.7)
Patient centered oral health care planning	0 (0)	1 (33.3)	1 (50)	4 (100)	3 (25)	6 (24)	2 (100)	1 (50)	1 (50)	3 (100)	2 (66.6)	4 (100)	3 (100)	55 (44.7)
Communication skills	1 (33.3)	1 (33.3)	2 (100)	2 (50)	2 (16.7)	10 (40)	2 (100)	1 (50)	0 (0)	3 (100)	1 (33.3)	4 (100)	2 (66.6)	54 (43.9)
Interdisciplinary management of the ageing individual	1 (33.3)	2 (66.6)	1 (50)	4 (100)	4 (33.3)	6 (24)	2 (100)	0 (0)	0 (0)	3 (100)	1 (33.3)	4 (100)	3 (100)	50 (40.7)
Endodontic assessment	0 (0)	3 (100)	0 (0)	1 (25)	6 (50)	3 (12)	1 (50)	1 (50)	1 (50)	2 (66.6)	0 (0)	1 (25)	1 (33.3)	43 (35.0)
Oral health education on an individual and community based level	0 (0)	1 (33.3)	1 (50)	3 (75)	3 (25)	6 (24)	1 (50)	0 (0)	0 (0)	3 (100)	1 (33.3)	2 (50)	2 (66.6)	43 (35.0)
Legal issues (ie. Ability to consent)	1 (33.3)	1 (33.3)	1 (50)	3 (75)	3 (25)	2 (8)	2 (100)	0 (0)	0 (0)	3 (100)	1 (33.3)	4 (100)	1 (33.3)	37 (30.1)
Principles and practice of domiciliary care	1 (33.3)	0 (0)	1 (50)	3 (75)	3 (25)	4 (16)	1 (50)	1 (50)	0 (0)	3 (100)	0 (0)	4 (100)	1 (33.3)	35 (28.5)
Recording history	0 (0)	2 (66.6)	1 (50)	3 (75)	0 (0)	5 (20)	2 (100)	0 (0)	0 (0)	3 (100)	0 (0)	2 (50)	2 (66.6)	34 (27.6)
Principles and practice of palliative care	1 (33.3)	0 (0)	1 (50)	3 (75)	2 (16.7)	2 (8)	1 (50)	0 (0)	0 (0)	3 (100)	0 (0)	4 (100)	2 (66.6)	27 (22)

*n* number of schools

^a^Percentages calculated based on the number of respondent schools per country

^b^Percentages calculated based on the number of all respondent schools (*n* = 123)

The most common educational technique utilized was didactic lectures (84 schools, 68.3%). Other educational methods reported were small group seminars (34 schools, 27.6%), problem-based learning (22 schools, 17.9%), conducting research (9 schools, 7.3%), maintaining student portfolios (10 schools, 8.1%), blended learning (combination of e-learning and face-to-face learning) (6 schools, 4.9%), and e-learning (4 schools, 3.3%).

Clinical teaching in Gerodontology was offered in 79 schools (64.2% of the respondents) and in 62 schools it was a compulsory element (50.4% of the respondents). In most schools clinical training was delivered within the dental school, usually embedded in other disciplines’ clinics or in Comprehensive Care Clinics (*n* = 47, 38.2%), and in fewer schools in a dedicated Gerodontology clinic (*n* = 11, 8.9%) or in both (*n* = 3, 2.4%). A total of 33 (26.8%) schools reported clinical training in outreach facilities, mainly nursing homes (16.3%), geriatric hospitals (10.6%), patients’ homes (5.7%) and geriatric day centers (4.1%). One school had a mobile unit. Sixteen schools (13%) offered clinical training only in outreach facilities, 16 schools (13.0%) offered training in the both dental school and outreach, whilst 43 schools (35.0%) offered clinical training only within the dental school. A few schools did not provide more details on their clinical program.

A large number of dental procedures were performed during clinical teaching (Table 3). The most frequent elements recorded were “oral health prevention and education”, “denture assessment, repair and/or fabrication” and “oral health care plans” (Table 3). In outreach training the most common dental procedures were “oral health prevention and education” and “oral health care plans”, while a large variety of dental procedures was offered to older patients treated within the dental schools (in Gerodontology clinics, in various disciplines’ clinics or in Comprehensive Care Clinics).

**Table 3:** The content of undergraduate clinical teaching in Gerodontology in selected countries with high response rates (>70%) and in all respondent schools

Content of clinical teaching	Austria n (%)^a^	Croatia n (%)^a^	Denmark n (%)^a^	Finland n (%)^a^	France n (%)^a^	Germany n (%)^a^	Greece n (%)^a^	Ireland n (%)^a^	Lithuania n (%)^a^	Norway n (%)^a^	Sweden n (%)^a^	Switzerland n (%)^a^	Netherlands n (%)^a^	Total number of schools n (%)^b^
Oral health prevention and education	2 (66.7)	2 (66.7)	1 (50)	4 (100)	6 (50)	9 (36)	2 (100)	2 (100)	1 (50)	3 (100)	1 (33.3)	4 (100)	1 (33.3)	68 (55.3)
Dentures assessment repairs and/or fabrication	2 (66.7)	2 (66.7)	1 (50)	1 (25)	5 (41.7)	9 (36)	2 (100)	2 (100)	1 (50)	3 (100)	1 (33.3)	4 (100)	1 (33.3)	62 (50.4)
Oral health care plan	1 (33.3)	2 (66.7)	1 (50)	4 (100)	5 (41.7)	8 (32)	2 (100)	1 (50)	1 (50)	3 (100)	1 (33.3)	3 (75)	1 (33.3)	60 (48.8)
Caries management	0 (0)	2 (66.7)	1 (50)	1 (25)	5 (41.7)	7 (28)	2 (100)	2 (100)	1 (50)	3 (100)	1 (33.3)	3 (75)	0 (0)	55 (44.7)
Periodontal treatment	0 (0)	2 (66.7)	1 (50)	0 (0)	5 (41.7)	7 (28)	2 (100)	2 (100)	1 (50)	3 (100)	1 (33.3)	2 (50)	0 (0)	55 (44.7)
Teeth extractions	0 (0)	2 (66.7)	1 (50)	1 (25)	5 (41.7)	7 (28)	2 (100)	2 (100)	1 (50)	3 (100)	1 (33.3)	3 (75)	0 (0)	54 (43.9)
Follow-up and recall	1 (33.3)	2 (66.7)	1 (50)	1 (25)	4 (33.3)	8 (32)	2 (100)	1 (50)	1 (50)	3 (100)	1 (33.3)	2 (50)	1 (33.3)	51 (41.5)
Fixed Prosthodontics	1 (33.3)	2 (66.7)	1 (50)	0 (0)	4 (33.3)	7 (28)	2 (100)	2 (100)	0 (0)	3 (100)	1 (33.3)	1 (25)	0 (0)	44 (35.8)
Endodontic treatment	0 (0)	2 (66.7)	0 (0)	0 (0)	5 (41.7)	4 (16)	2 (100)	1 (50)	1(50)	3 (100)	1 (33.3)	0 (0)	0 (0)	41 (33.3)
Implants provision	0 (0)	2 (66.7)	0 (0)	0 (0)	3 (25)	6 (24)	0 (0)	1 (50)	0 (0)	0 (0)	1 (33.3)	1 (25)	0 (0)	21 (17.1)

*n* number of schools

^a^Percentages calculated based on the number of respondent schools per country

^b^Percentages calculated based on the number of all respondent schools (*n* = 123)

The most common format of educational material was PowerPoint presentations (90 schools, 73.2%), followed by scientific articles (59 schools, 48%), printed textbooks (53 schools, 43.1%), lecture notes (48 schools, 39%), e-learning material (26 schools, 21.1%), videos (18 schools, 14.6%), and e-books (9 schools, 7.3%).

Forty-four schools (35.8%) mentioned that they are familiar with the ECG undergraduate curriculum guidelines.

### Postgraduate gerodontology education

A total of 38 European schools (30.9%) included postgraduate Gerodontology teaching in their curricula, usually embedded in other specialty programs (23 schools, 18.7%). Eleven schools offered Gerodontology training in multiple disciplines. Similar to the undergraduate teaching, postgraduate Gerodontology training was mainly included in the Prosthodontics courses (19 schools, 15.5%) (Table 4). Postgraduate courses, exclusively dedicated to Gerodontology, were only offered in 9 schools (7.3%), usually as distinctive components of other specialty programs. However, a significant number of schools did not specify the affiliation details.

**Table 4:** Disciplines that teach Gerodontology topics at the postgraduate curricula in selected counties with high response rates (>70%) and in all respondent schools

Disciplines	Croatia n (%)^a^	Finland n (%)^a^	France n (%)	Germany n (%)^a^	Greece n (%)^a^	Norway n (%)^a^	Sweden n (%)^a^	Switzerland n (%)^a^	Total number of schools (%)^b^
Prosthodontics	1 (33.3)	1 (25)	1 (8.3)	3 (12)	2 (100)	2 (66.7)	1 (33.3)	3 (75)	19 (15.4)
Periodontology	0 (0)	0 (0)	1 (8.3)	1 (4)	0 (0)	2 (66.7)	0 (0)	0 (0)	7 (5.7)
Oral and Maxillofacial Surgery	0 (0)	0 (0)	0 (0)	2 (8)	0 (0)	2 (66.7)	0 (0)	1 (25)	6 (4.9)
Special Care Dentistry	0 (0)	0 (0)	1 (8.3)	0 (0)	0 (0)	1 (33.3)	0 (0)	0 (0)	4 (3.3)
Oral Pathology	0 (0)	0 (0)	1 (8.3)	0 (0)	0 (0)	1 (33.3)	0 (0)	0 (0)	3 (2.4)
Preventive and Community Dentistry	0 (0)	0 (0)	0 (0)	0 (0)	0 (0)	0 (0)	0 (0)	0 (0)	3 (2.4)
Operative Dentistry	0 (0)	0 (0)	0 (0)	0 (0)	0 (0)	2 (66.7)	0 (0)	0 (0)	2 (1.6)
Endodontics	0 (0)	0 (0)	0 (0)	0 (0)	0 (0)	1 (33.3)	0 (0)	0 (0)	1 (0.8)
Master in Dentistry research	0 (0)	0 (0)	0 (0)	0 (0)	0 (0)	0 (0)	0 (0)	0 (0)	1 (0.8)
Restorative Dentistry	0 (0)	0 (0)	0 (0)	0 (0)	0 (0)	0 (0)	0 (0)	0 (0)	1 (0.8)

*n* number of schools

^a^Percentages calculated based on the number of respondent schools per country

^b^Percentages calculated based on the number of all respondent schools (*n* = 123)

Twenty of the 84 schools (23.8%) currently not offering postgraduate Gerodontology teaching, indicated that they were currently considering offering a program in the near future.

The western European countries recorded the highest activity rates (38.8%) whilst the Eastern European countries recorded the lowest ones (15.4%) (Fig. 1).

### Continuing education (continuing professional development/CPD) in gerodontology offered by dental schools

A total of 37 schools (30.1%) reported that they currently offered continuing education courses in Gerodontology. The Northern European countries recorded the highest rates of teaching activity (35.7%) and the Eastern European countries the lowest rates (23.1%) (Fig. 1).

### Educational material

Eighty-eight schools (71.5%) claimed that Gerodontology educational material was developed and published in their country. Respondents listed textbooks, scientific articles, lecture notes, e-learning material and videos, as examples of the education materials.

## Discussion

The findings of the present survey indicated that the vast majority of European dental schools who responded to this survey teach Gerodontology at the undergraduate level (86.2%), whilst smaller numbers offer teaching at the postgraduate and continuing education levels.

The response rate to this electronic questionnaire was 56.9%, which is considered satisfactory for an online-survey, delivered in 39 different countries, speaking different languages. One important limitation was that the questionnaire was only offered in English and this may have been a significant barrier for many countries where English is not widely spoken. As 43.1% of the schools did not respond to the survey, a worst case scenario should be kept in mind, where the non-responding schools may not teach Gerodontology. Moreover, no complete list of European dental schools was available, hence some may have not been included. As for the activity levels of continuing education courses reported in this study, these should be interpreted with caution, as in addition to Universities, there is a vast number also on offer from both commercial providers and learned societies [18].

### Comparison with previous studies in Europe

Previous data on Gerodontology teaching in Europe are limited. There is only one study conducted in 2002, with data from 82 schools in 27 countries (42.0% response rate) [17]. Even if the low response rate in the 2002 study and differences in participating countries and schools may preclude direct comparisons, some interesting observations can still be made. When comparing the 2002 data with our findings, it seems that in the past 14 years a decreasing number of schools teach Gerodontology at the undergraduate level (−7.6%), there are fewer dedicated Gerodontology clinics (−6.6%), and fewer mandatory courses (−14.5%). The crowded European curricula promoting elective courses and the tendency for having Comprehensive Care Clinics may have contributed to for this change. Positive changes are the slight increases in the dedicated Gerodontology courses (+1.4%), in clinical training (+3.2%) and in the visitations in nursing homes (+3.3%) when compared to the survey from Preshaw et al. [19]. Significant positives include the increase by 16% of the assignment of Gerodontology program directors and the increase by 35% in the training of the program directors in Gerodontology.

A number of regional and national studies have been previously published, showing that in some countries there has been a long tradition in Gerodontology teaching. In German-speaking countries (Germany, Austria and Switzerland) an increase in undergraduate Gerodontology teaching from 2004 to 2009 was recorded [19], including dedicated Gerodontology courses [20]. In Athens, Greece, Gerodontology education has been developed within Removable Prosthodontics since 1991, and has been taught as a dedicated course since 2003 [21]. The 2004–2006 dental schools’ graduates in Belgium reported increased variation in the Gerodontology curricula among the Belgian dental schools [22], while in Spain 42.0% of schools offered a specific Gerodontology course in 2015, with only one school offering training in outreach settings [23].

### Comparison with gerodontology teaching in other continents

There is limited information regarding Gerodontology teaching outside Europe.

Information is available for the United States of America (USA) dental schools, showing a long tradition in teaching, facing similar weaknesses as Europe, mainly related to the limited clinical training in frail and medically compromized older people [24], and the variation in the organization, structure, amount and content of the Gerodontology curriculum [25]. Both a survey published in 2003 [16] and a web-search study in 2013 [24] have shown that almost all USA dental schools teach at least some aspects of Gerodontology at the predoctoral level. A total of 98.0% of the schools had a compulsory didactic element, 63.0% had a Geriatric program director or a chairman of a Geriatric section, half of the schools had a dedicated lecture course and 67.0% offered a clinical component [16]. Regarding postgraduate training, 69.0% of the USA schools included a Geriatric component in either their General Practice Residency, Advanced Education in General Dentistry program or offer a certificate program in Geriatric Dentistry [24], compared to 31.0% in Europe. However only 9 schools (15%) offered a certificate or fellowship in Gerodontology. On the other hand, 23.0% of the USA schools offered a continuing education course in Dental Geriatrics at any one time [24] compared to 30.1% recorded in Europe.

A 2010 study in Japan, the country with the largest proportion of elders in the world, revealed that 10 out of the 29 Japanese dental schools had specific Gerodontology departments, while the others taught Gerodontology within Prosthodontics [26]. In the same schools a 4-year postgraduate PhD course was offered, but masters and certificate programs were not available [26].

Brazil recognized Gerodontology as a specialty in 2001, but until December 2015 only 275 dentists had successfully graduated, while very few schools reported teaching Gerodontology at the undergraduate level, mainly within Prosthodontics [27, 28]. A web-survey in Chile has shown that 84% of the dental schools taught some aspects of Gerodontology in the undergraduate curricula [29], while in Australia Gerodontology is not currently a significant component of dental curricula [30].

### Implementation of the ECG curriculum guidelines

In order to fully implement the ECG undergraduate curriculum guidelines [12] in European dental schools progress is still required. The ECG guidelines recommend that Gerodontology should be mandatory, run by a Gerodontology department or division or by a group of teachers specifically assigned to the course. The present survey shows that Gerodontology was mandatory in only 67.5% of the schools, a number not meeting the ECG recommendations. Almost 44% of the respondents has a dedicated program director, but fewer had a specific Gerodontology Unit (17.1%). The ECG guidelines emphasize the close integration of Gerodontology with Medicine and the necessity of an interdisciplinary educational team, but this was reported by only 26.8% of the schools. Furthermore, the interdisciplinary management of the ageing individual, as a theoretical topic, was taught in 40.7% of the schools. The ECG guidelines also recommend that training be integrated vertically throughout the curriculum, with theoretical information offered in the pre-clinical years, and clinical training when related to frail elders offered to senior students. This survey has shown that only 18.0% of the schools have integrated Gerodontology throughout the curriculum or in modules (if they follow a modular curriculum), while clinical training was offered in 64.2% of the schools.

Regarding the Gerodontology theoretical content, there was a significant agreement (over 50%) for issues identified in the ECG guidelines. These topics included biology and pathology of aging, association of general with oral health, demographics, oral health epidemiology, nutritional and chewing problems, while dental conditions that were very commonly taught were xerostomia, prosthodontic management, and caries risk assessment. More variability was recorded for clinical training, as 38.2% of schools have embedded Gerodontology training in other disciplines’ clinics or in Comprehensive Care Clinics, therefore a large variability of procedures was offered to community-dwelling elders.

The emphasis on Prosthodontics may not come as a surprise, as almost 31% of schools have embedded Gerodontology in the Prosthodontics departments, while 63.4% of the educators were prosthodontists. Complete and partial edentulism, are still very common in old age, while most of the patients requiring removable dentures are older, often medically compromised, and require prosthodontic techniques that enhance adaptation and motor coordination. Recent data from the German national survey (DMS V) confirms that 72.8% of persons aged 75 years and over were wearing removable dentures [31], while in Switzerland the prevalence of removable prostheses in persons aged over 85 years was 85.9% [32].

The EGG teaching guidelines emphasized the importance of students’ training in treatment planning and clinical care for older people with various levels of dependency, including the frail and functionally dependent ones. This is becoming very important considering the increasing numbers of the “oldest old” persons in Europe who should be primarily assessed and managed by general dental practitioners and referred to specialists or hospital dentists only when necessary. Apart from the appropriate theoretical training, clinical training in outreach facilities (community settings, nursing homes, private homes, and geriatric hospitals), offered mainly to senior students with adequate competences in General Medicine and Dentistry, would improve their competencies in managing the frail and dependent patients. However, this survey has identified limited relevant training opportunities as indicated by the limited training in outreach locations by only 26.8% of schools, the few interdisciplinary courses offered, the small number of schools teaching principles and practice of domiciliary care, and the few dedicated Gerodontology clinics.

### Barriers and opportunities for gerodontology teaching in Europe

It is recognized that most schools face significant barriers to implement the ECG guidelines for Gerodontology teaching, including a busy curriculum, lack of sufficiently trained staff and resources and faculty members with other teaching priorities [20, 21, 24, 29, 33, 34].

In the present survey, less than half (46.3%) of the program directors had received training in Gerodontology or Special Care Dentistry. Even if the proportion of trained educators in Gerodontology has increased in the last decade, a larger number of training opportunities for faculty are imperative. However, Gerodontology is not currently a recognized specialty in Europe, Canada or in the USA and advanced training opportunities are limited worldwide. In 2016, a framework for core competencies in Geriatric Dentistry Fellowship programs in the USA was proposed, based on the high demand for educating both dental practitioners and academics in the country [35]. The development of a European postgraduate core curriculum in Gerodontology that will be the basis for the development of Gerodontology Specialty programs in European dental schools is necessary.

It is noteworthy that a large proportion of the dental schools that did not currently teach Gerodontology at the undergraduate level (47.1%), reported plans to undertake teaching in the near future, while 23.8% of those without postgraduate Gerodontology teaching also considered beginning soon. Considering the barriers, already reported worldwide, these schools may need support in developing these courses. Dental schools with limited resources in faculty and educational material may benefit from the use of ICT (Information and Communication Technologies), including webinars, virtual patents’ cases, Massive Open Online Courses (MOOCs) and other e-learning resources. The development of standardized electronic teaching modules, according to agreed competences, is a necessary initiative. However, the current survey revealed that few schools use new technologies in Gerodontology teaching, and they largely rely on traditional techniques such as lecturing.

Educational collaborations, exchange of students, faculty and educational material among European dental schools, offer a great opportunity for further development and convergence of Gerodontology teaching in the continent.

Finally, as Gerodontology is closely related to Medicine and auxiliary health professions, the development of interprofessional education programs where dentists, dental hygienists, physicians, nurses, pharmacists, occupational therapists, physical therapists, social workers and other care providers working with older people, learn together and from each other, offer significant educational opportunities for Gerodontology training for students, dental faculty and practicing dentists [36, 37].

## Conclusions

Within the limitations of the present study, a large proportion of European dental schools (86.2%) teach Gerodontology at the undergraduate level. More training opportunities in the oral care of frail and dependent older people should be offered, and more emphasis should be placed on interdisciplinary teaching and interprofessional training, educational collaborations and the use of modern technologies. A small number of schools (31.0%) offer Gerodontology postgraduate training, mostly embedded in Prosthodontics courses. Dedicated postgraduate Gerodontology courses need to be developed to create a significant number of specialized dentists and trained academics. A total of 30.1% of European dental schools already offer Gerodontology training at the continuing education level, reflecting the increasing demand for training of the active dental workforce.

The continued dissemination of the ECG undergraduate curriculum guidelines will help to increase teaching activity and improve the standard of Gerodontology teaching in Europe.

## Additional file

Additional file 1: Questionnaire for Gerodontology education in Europe: the pdf format of the electronic questionnaire used in the present study. (PDF 392 kb)

## Abbreviations

ADEE: Association for Dental Education in Europe
CE: Continuing education
CED: Council of the European dentists
CPD: Continuing professional development
ECG: European College of Gerodontology
ICT: Information and communication technologies
MOOCs: Massive open online courses
