# Supplementary material for: Higher education in Gerodontology in European Universities
Source: BMC Oral Health. 2017 Mar 28;17:71. doi: 10.1186/s12903-017-0362-9 (PMC5371193; doi:10.1186/s12903-017-0362-9)
Supplement: Additional file 1: — Questionnaire for Gerodontology education in Europe: the pdf format of the electronic questionnaire used in the present study. (PDF 392 kb) [file 12903_2017_362_MOESM1_ESM.pdf]

Additional file 1

Survey questionnaire for the current status of Gerodontology education in Europe

(pdf format of the electronic questionnaire developed by A. Kossioni et al.)

GENERAL INFORMATION

1. **Full name of the University / Dental School**
2. **Country**
3. **Name, position and email address of the dental school's contact person**
4. **Telephone of the dental school's contact person**
5. **Website of the dental school**
6. **How many years is the duration of the undergraduate dental studies at your dental school?**
   - Four years
   - Five years
   - Six years
   - Other (please explain)….
7. **Is your dental school public or private?**
   - Public
   - Private
   - Other (please explain)….
8. **Official teaching language at your dental school** ….
9. **How many dental schools are there in your country?**
10. **Is Gerodontology a recognized specialty in your country?**
    - Yes
    - No
    - Don't know
    - Other (please explain)….
11. **Are there any dentists specialising in Gerodontology in your country?**
    - Yes
    - No
    - Don't know
    - Other (please explain)….
12. **Is there a Gerodontology Scientific Association in your country?**
    - Yes
    - No
    - Don't know
    - Other (please explain)….

©2016 Anastassia Kossioni, Gerry McKenna, Frauke Müller, Martin Schimmel, and Jacques Vanobbergen

If yes,

13. **If you can, please provide the name of the Gerodontology Association in your country**
14. **If you can, please provide the web address of the Gerodontology Association in your country**

UNDERGRADUATE GERODONTOLOGY TEACHING

15. **Is Gerodontology being taught at the undergraduate studies?**
    - Yes
    - No
    - Other (please explain)….

If no,

16. **Do you consider starting undergraduate Gerodontology teaching soon?**
    - Yes
    - No
    - Don't know
    - Other (please explain)….
17. **Is there a specific Gerodontology Department/Division/Unit at your dental school?**
    - Yes
    - No
    - Other (please explain)….

If yes,

18. **Please type the name of the Gerodontology Department/Division/Unit in your own language**
19. **Please type the name of the Gerodontology Department/Division/Unit in English**
20. **Which is the Department/Division/Unit of the Programme Director?**
    - Gerodontology
    - Special Care Dentistry
    - Prosthodontics
    - Preventive and Community Dentistry
    - Other (please explain)….
21. **What is the formal training of the Gerodontology Programme Director?**
    - Gerodontology
    - Special Care Dentistry
    - Prosthodontics
    - Preventive and Community Dentistry
    - Other (please explain)….

©2016 Anastassia Kossioni, Gerry McKenna, Frauke Müller, Martin Schimmel, and Jacques Vanobbergen

22. **Is Gerodontology an elective or a compulsory (required) course according to your curriculum?**
    - Elective
    - Compulsory (required)
    - Only some parts of the course are compulsory (ie. the theoretical component).
23. **If only some parts of the course are compulsory, please offer more details….**
24. **How many years is Gerodontology taught at the undergraduate curriculum at your dental school? …**
25. **Is Gerodontology taught as an independent course?**
    - Yes
    - No
    - Other (please explain)….
26. **Is Gerodontology teaching integrated in other disciplines' courses?**
    - Yes
    - No
    - Other (please explain)….

If yes,

27. **Which discipline(s) include Gerodontology teaching?**
    - Prosthodontics
    - Preventive and Community Dentistry
    - Special Care Dentistry
    - Operative Dentistry
    - Periodontology
    - Oral and Maxillofacial Surgery
    - Oral Pathology
    - Endodontics
    - Other (please explain)….
28. **What is the composition of the Gerodontology Educators?**
    - Dentists
    - Physicians
    - Nurses
    - Psychologists
    - Social workers
    - Other (please explain)….

©2016 Anastassia Kossioni, Gerry McKenna, Frauke Müller, Martin Schimmel, and Jacques Vanobbergen

29. **What are the teaching disciplines of the Dental Educators in the Gerodontology course?**

- Gerodontology
- Prosthodontics
- Special Care Dentistry
- Preventive and Community Dentistry
- Operative Dentistry
- Periodontology
- Oral and Maxillofacial Surgery
- Endodontics
- Other (please explain)….

30. **In which semester(s) is Gerodontology taught? (If your school offers an introductory year, then the 1st semester is the one officially starting the dental studies)**

- 1st semester
- 2nd semester
- 3rd semester
- 4th semester
- 5th semester
- 6th semester
- 7th semester
- 8th semester
- 9th semester
- 10th semester
- 11th semester
- 12th semester
- Other (please explain)….

31. **Does Gerodontology teaching have a theoretical component?**

- Yes
- No
- Other (please explain)….

©2016 Anastassia Kossioni, Gerry McKenna, Frauke Müller, Martin Schimmel, and Jacques Vanobbergen

32. **How is theoretical training delivered?**

- Lectures
- Small group seminars
- Problem-based teaching
- Occasional lectures throughout the curriculum
- Information embedded in other courses' lectures and seminars
- E-learning
- Blended learning (e-learning + face to face teaching)
- Research projects
- Other (please explain)….

33. **What are the theoretical topics in Gerodontology teaching at your school?**

- Demographics
- Barriers to oral care
- Biology, physiology, psychology of ageing
- Medical problems in old age
- Pharmacology and polypharmacy in old age
- Association between general and oral health in the elderly population
- Epidemiology of oral health in the elderly population
- Age-changes of the orofacial system
- Communication skills
- Nutritional/chewing problems in old age
- Interdisciplinary (interprofessional) management of the ageing individual
- Legal issues (ie. ability to consent)
- Ethical aspects
- Recording history
- Risk assessment in the ageing individual
- Salivary impairment/ xerostomia
- Periodontal disease in old age
- Denture related conditions and prosthodontic management in old age
- Caries risk assessment, particularly root caries
- Tooth wear assessment
- Endodontic assessment
- Oral mucosal diseases in old age
- Patient centered oral health care planning

©2016 Anastassia Kossioni, Gerry McKenna, Frauke Müller, Martin Schimmel, and Jacques Vanobbergen

- Appropriate management of oral and dental conditions for each patient according to the individual needs and demands
- Management of people with compromised health and various levels of dependency
- Principles and practice of domiciliary care
- Principles and practice of palliative care
- Oral health education on an individual and community based level
- Other (please explain)….

34. **Does Gerodontology teaching have a pre-clinical component?**
    - Yes
    - No
    - Other (please explain)….
35. **If your undergraduate Gerodontology curriculum includes a pre-clinical component, please specify….**
36. **Does Gerodontology teaching have a clinical component?**
    - Yes
    - No
    - Other (please explain)….

If, yes:

37. **Is clinical training compulsory or elective?**
    - Compulsory
    - Elective
    - Other (please explain)….
38. **Where is clinical Gerodontology training delivered?**
    - In the Dental School, in a dedicated Gerodontology clinic
    - In the Dental School, embedded in other disciplines' clinics
    - In Geriatric Hospitals
    - In Nursing homes
    - In Older people Day Centers
    - In patients' houses after house calls (Domiciliary Care)
    - In Community clinics
    - In Mobile units
    - Other (please explain)….

©2016 Anastassia Kossioni, Gerry McKenna, Frauke Müller, Martin Schimmel, and Jacques Vanobbergen

39. **What is the content of Gerodontology clinical training?**

- Oral health care plan
- Dentures assessment, repairs and/or fabrication
- Fixed Prosthodontics provision
- Oral health prevention and education
- Caries management
- Endodontic treatment
- Periodontal treatment
- Dental implants provision
- Teeth extractions
- Follow-up and recall
- Other (please explain)….

40. **Are you familiar with the European College of Gerodontology (ECG) undergraduate curriculum guidelines (Gerodontology 2009;26(3):165-71)?**

- Yes
- No
- Other (please explain)….

41. **What type of educational material do you use in Gerodontology teaching?**

- PowerPoint Presentations
- Video display
- E-learning material
- Portfolio
- Webinars
- Lecture Notes
- Printed Textbooks
- E-Books
- Scientific Articles
- Other (please explain)….

POSTGRADUATE GERODONTOLOGY TEACHING

42. **Is Gerodontology being taught in the postgraduate curriculum at your dental school?**

- Yes
- No
- Other (please explain)….

©2016 Anastassia Kossioni, Gerry McKenna, Frauke Müller, Martin Schimmel, and Jacques Vanobbergen

If no

43. **Do you consider starting Gerodontology teaching at the postgraduate curriculum soon?**
    - Yes
    - No
    - Don’t' know
    - Other (please explain)….
44. **Is there a dedicated Gerodontology postgraduate course offered at your dental school?**
    - Yes
    - No
    - Other (please explain)….
45. **Is postgraduate training in Gerodontology offered at your dental school embedded in other postgraduate courses?**
    - Yes
    - No
    - Other (please explain)….

    If yes,
46. **If Gerodontology is embedded in other postgraduate courses, please specify:**
    - Prosthodontics
    - Special Care Dentistry
    - Preventive and Community Dentistry
    - Operative Dentistry
    - Periodontology
    - Oral Pathology
    - Oral and Maxillofacial Surgery
    - Endodontics
    - Other (please explain)….
47. **Do you run any Continuing Education Courses in Gerodontology at your dental school?**
    - Yes
    - No
    - Other (please explain)….

©2016 Anastassia Kossioni, Gerry McKenna, Frauke Müller, Martin Schimmel, and Jacques Vanobbergen

GERODONTOLOGY EDUCATIONAL MATERIAL

48. **Is there any Gerodontology educational material developed and published in your country (eg. textbooks, lecture notes, e-learning material etc)?**
    - Yes
    - No
    - Don't know
    - Other (please explain)….
49. **If you wish, please add more details about the Gerodontology educational material published in your country…**

ADDITIONAL DETAILS

50. **Please provide your role and title (Head/Faculty/Administration, etc) ….**
51. **If you wish, please add any additional thoughts or comments on the questionnaire…..**
52. **If you wish, please provide the name, title and email address of the Faculty/Educator responsible for Gerodontology teaching at your dental school to facilitate communication with the European College of Gerodontology…..**

Many thanks for taking the time to complete this questionnaire

©2016 Anastassia Kossioni, Gerry McKenna, Frauke Müller, Martin Schimmel, and Jacques Vanobbergen
